# Tracing Copper Migration in the Tongling Area through Copper Isotope Values in Soils and Waters

**DOI:** 10.3390/ijerph15122661

**Published:** 2018-11-27

**Authors:** Jingwen Su, Ryan Mathur, Glen Brumm, Peter D’Amico, Linda Godfrey, Joaquin Ruiz, Shiming Song

**Affiliations:** 1 Chinese Geological Survey, Nanjing Center, Nanjing 210016, China; jwsu@sina.com (J.S.); njumao@163.com (S.S.); 2 Geology Department, Juniata College, Huntingdon, PA 16652, USA; brummgx15@juniata.edu (G.B.); DAMICPA16@juniata.edu (P.D.); 3 Department of Earth and Planetary Sciences, Rutgers University, New Brunswick, NJ 08854, USA; linda.v.godfrey@gmail.com; 4 Department of Geosciences, University of Arizona, Tucson, AZ 85721, USA; jruiz@geo.arizona.edu

**Keywords:** copper isotopes, Tongling, copper concentration, mining

## Abstract

Copper mining in Tongling has occurred since the Bronze Age, and this area is known as one of the first historic places where copper has been, and is currently, extracted. Multiple studies have demonstrated, through concentrated work on soils and waters, the impact of mining in the area. Here we present copper isotope values of 13 ore samples, three tailing samples, 20 water samples (surface and groundwater), and 94 soil samples (15 different profiles ranging in depth from 0–2 m) from proximal to distal (up to 10 km) locations radiating from a tailings dam and tailings pile. Oxidation of the copper sulfide minerals results in isotopically heavier oxidized copper. Thus, copper sourced from sulfide minerals has been used to trace copper in mining and environmental applications. At Tongling, higher copper isotope values (greater than 1 per mil, which are interpreted to be derived from copper sulfide weathering) are found both in waters and the upper portions of soils (5–100 cm) within 1 km of the source tailings. At greater than 1 km, the soils do not possess heavier copper isotope values; however, the stream water samples that have low copper concentrations have heavier values up to 6.5 km from the source. The data suggest that copper derived from the mining activities remains relatively proximal in the soils but can be traced in the waters at greater distances.

## 1. Introduction

Tracing the release of metals due to mining practice is an essential activity to understand the overall ecological well-being of an area. Chemical analyses of all components of the biosphere have been used to assess the potential degradation of an area. Tongling has been an active mining area of a copper skarn deposit for over 3000 years, and multiple studies have shown metal dispersion related to mining with both concentration and isotope data from soils, waters, aerosols, and plants [1,2,3].

**Figure 1:**
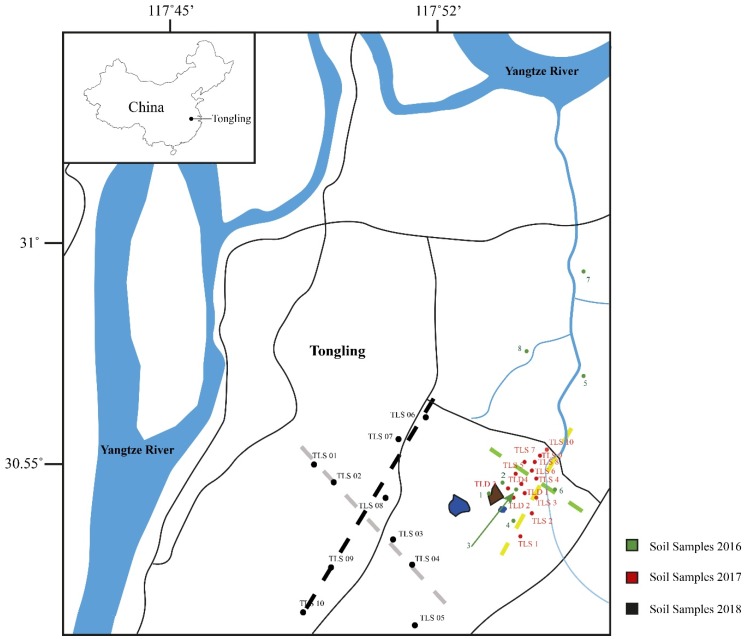
Field area with soil sample locations as colored circles. Dashed lines indicate lines of cross sections, as seen in Figure 5, where the yellow line is Section 1, the green line is Section 2, black line is Section 3, and the grey line is Section 4. Dark lines indicate smaller streams found along the roadside in the area. The blue ‘lake’ like symbol indicates the position of the tailings dam, and the dark colored square indicates the older tailing pile location.

Here, we use copper isotope compositions of soils and waters to trace Cu migration from an actively weathering tailings pile, along with assessing airborne contaminates (derived from a prevailing northwesterly wind) from a metal processing plant in Tongling (Figure 1). Transition metal and metal isotope geochemistry are relatively new techniques that have been applied to understand the rates, dispersion, and ability to trace sources of metal release induced by surface and subsurface mining activities. It has been shown that copper isotope values can provide key insights into the kinetics of the reactions related to weathering and dispersion of Cu due to oxidative destruction of sulfide minerals [4,5,6,7,8,9], along with tracing airborne contaminants from smelters and mine tailings [10,11]. These studies have demonstrated that the copper isotope compositions of different components in the biosphere are highly sensitive to inputs of metal from different contamination sources. Daily changes in the copper isotope compositions of soils and waters can be related to isotopically unique inputs [6,12]. What is apparent from these studies is that the copper isotope compositions of waters and soils quickly respond to changes in sources of copper on the orders of days to weeks. Thus, the rates of weathering of exposed sulfide minerals control the ability to trace mining activities from different times in the past.

**Figure 2:**
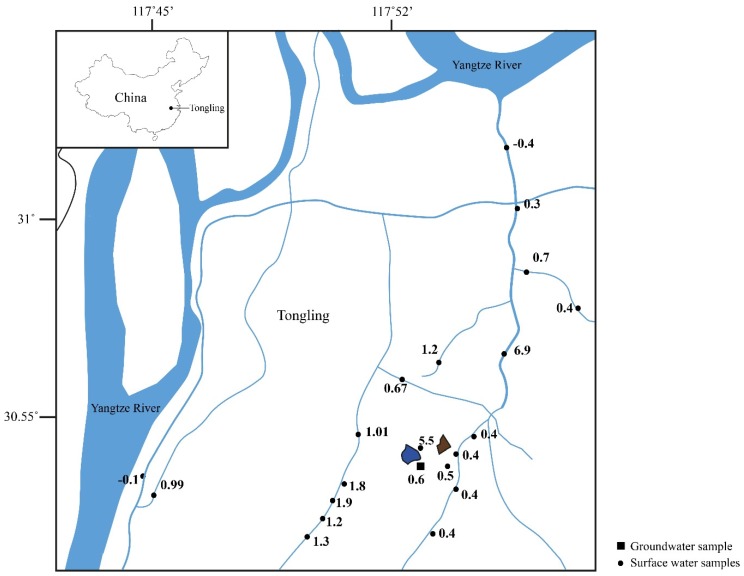
Water sample locations with labeled copper isotope values measured at each location. Blue lake near the groundwater sample is the current tailings impoundment, and the brown triangular symbol indicates tailing location.

The current challenges for this science reside in understanding the complicated pathways dissolved copper can experience once released into the biosphere. Both experimental and empirical datasets have demonstrated that copper isotope compositions of materials are changed by both mineralogical [13,14,15,16,17] and biological processes [18,19,20]. Thus, interpretations of the copper isotope data must account for the pathways copper could experience. The sampling in Tongling presents a new case for observation of Cu isotopes in acid mine drainage (AMD) impacting surface waters and soils (ferasols) in a highly buffered karst environment. The objective of the project is to assess metal migration from the Tongling mining activities into the Yangtze drainage basin. Sampling of soils and waters across drainage divides and into the Yangtze River were analyzed to accomplish this goal (Figure 1 and Figure 2).

Elevated isotope values found in soils and waters identify copper sourced from weathered sulfides (ore related products). Several studies have clearly demonstrated that oxidation of sulfide minerals during weathering results in the release of heavy copper into solution [7,17,21]. The heavier copper has a distinct signature from weathering of silicate minerals and other potential sources of copper. To map the lateral and vertical extent of contamination, both plan view plots of the water data (Figure 2) and cross section plots of the soil data (Figure 1) are used to identify the extent of this point source contamination.

## 2. Materials and Methods

The sampling campaign spanned a 3-year interval. Due to the multiple point sources for metal (weathering of tailings, tailings impoundment, and airborne material from the mineral processing plant), several sampling strategies were employed to understand the copper isotope composition of the source materials associated with their distribution in the area (Figure 1).

Weathered tailings and ores exposed due to mining are two of the sources of sulfides that provide significant amounts of copper. To characterize the sources, samples from the weathered tailings (brown polygon on Figure 1) and a drill core were taken from the active mining area. The weathered tailings samples were collected by augering into the tailings pile and sampling 3 depths in the hole. The material had both Fe-oxide minerals and partially weathered sulfide minerals. The sulfide minerals chosen were a mixture of sulfide phases in each sample. Essential to this study is the source of Cu, where it is predominantly found in the mineral chalcopyrite (CuFeS_2_). However, trace amounts of copper can be found in another more abundant sulfide, pyrite (FeS_2_). The mixture in the tailings samples were various proportions of these phases with pyrite > chalcopyrite. The unweathered ores, which represent the processed ores in the mill, were extracted from the drill core through hand-picking. A total of 9 chalcopyrite and 4 pyrite samples were selected to characterize these sources of copper in the ore deposit. Sulfide minerals were visually identified.

Soil samples were collected in close proximity to the point sources and systematically down hydrogeologic gradient and across the ridgeline (drainage divide) from the sources. The samples were intentionally collected to construct cross sections to allow determination of both lateral and vertical dispersion. Sample location 7, TSL5, and TSL 10 are comparison points that were not impacted by the mining activity.

Thirty-two holes were augered for soil sampling at depths which ranged from 0–300 cm. The varying depths of soil sampling allowed for the determining both lateral and vertical dispersion of copper in the system. None of the holes reached bedrock, even when sampling to greater than 2 m. Sampling depths for all locations were the first 10 cm; several holes were chosen for greater depths to provide a greater depth perspective closer to the mine site. Ninety-four total soil samples were collected. Soil samples were dried, powdered, and sieved to obtain homogenized soils samples of <50 micron particle size. The soils contained carbonates (calcite and siderite), Fe-oxide, Mn-oxides, and silicate clay minerals.

One groundwater and 19 surface water samples were collected in the field in 500 mL bottles. The groundwater sample was obtained at a depth of 2.5 m from a local water well. The water samples were filtered (200 micron filter) in the field, and then 50 mL of water was evaporated for chemical analysis. The temperatures (15–18 °C) and pHs (5.2–8.3) of the waters were recorded, and no patterns with these variables were noted.

The chemical purification of the soils and salts from the evaporated waters is relatively similar. For the soils, approximately 50 mg was dissolved in a heated (100 °C) Teflon beaker containing 5 mL of ultrapure 10N HF + 8N HNO_3_ + 5N HCl. Complete dissolution was visually confirmed. The solution was dried. The salts from the waters were dissolved in 3 mL of heated 16 N nitric acid to eliminate any potential organic material and then dried. After the dissolution steps, copper was isolated from the salts of the soils and waters with ion exchange chromatography using the Bio-Rad MP-1 resin chloride form with a 100–200 mesh size. The procedure is reported in References [14,22].

The purified salts were dissolved in 2% ultrapure nitric acid. The solutions were measured on ICP-MS multicollector mass spectrometers at the University of Arizona (Isoprobe Lab) and Rutgers University (Neptune Lab) between 2016 and 2018. Mass bias was corrected for by standard-sample-standard bracketing using the NIST 976 isotope standard, and values are reported relative to NIST 976 in the traditional per mil value (^65^Cu) given the following expression:

Cu65 ‰=((Cu65Cu63)sample(Cu65Cu63) NIST 976−1)∗1000

One peak background was subtracted for each value, and 1 block of 30 ratios occurred for each measurements. Samples were measured in duplicate, and reported values are the mean of the two measurements. The variation of the two values is less than that of the errors explained below.

The error for the sessions was calculated by bracketing the NIST 976 standard in comparison to the previous NIST 976 measurements. Because the standard had been measured the most times, the performance of the instrument was best understood by monitoring its variation throughout the session. The error of this value was 0.12‰ (2σ, n = 182) on the Isoprobe and 0.08‰ (2σ, n = 64) on the Neptune at Rutgers.

The quality of the data was monitored by measuring internal and international standards. An internal standard (1838 USA cent) was measured 14 times at each location and produced a value of −0.01 ± 0.08‰ (2σ), which is within the accepted value presented in Reference [23]. A US Geological Survey (USGS) rock standard (BVHO-2) was also monitored at each location during the measuring session and produced ^65^Cu values of 0.25 ± 0.09‰ (2σ, n = 5) at Arizona and 0.24 ± 0.05‰ (2σ, n = 3) at Rutgers, which are within the reported ranges of Reference [24].

## 3. Results

The sources of copper in the system have relatively tight ^65^Cu values (Table 1). The chalcopyrite ores have a mean value of −0.04 ± 0.26‰ (n = 9), and the pyrite is more variable with a mean value of 0.61 ± 0.94‰ (n = 4). The ranges of copper ores are in line with those previously reported for skarns and higher temperature mineralization systems as those present in Tongling [25,26,27,28,29,30]. The weathered tailings ^65^Cu values are lower (−0.34 to −0.66) than the starting ores and become higher with greater depth.

The waters show the greatest range of ^65^Cu values, from −0.13 to 6.90‰ (Table 2). The highest values measured occur near the mining activity (Figure 2). Beyond a 6 km radial distance from the mining activity, the values drop to below 0.4‰. The water well data point has a value of 0.6 per mil and is located 0.5 km down hydrogeologic gradient of the tailing dam.

**Figure 3:**
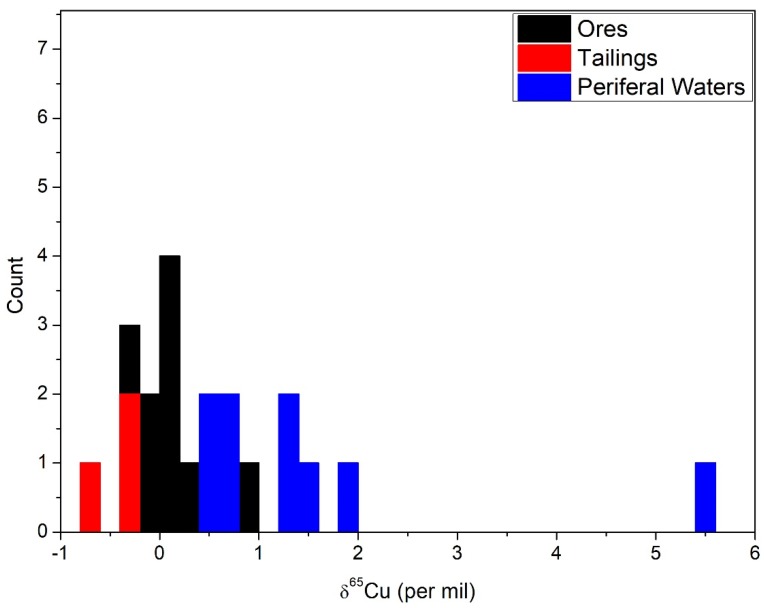
Frequency plot of the ores, tailings, and nearby surface waters which displays how weathered ores lose heavier copper that resides in the fluids which oxidized the sulfide minerals.

The frequency plot (Figure 3) of the ores, tailings, and waters display an important relationship. Notice that the ores center close to the zero value, and that the tailings are lower and the waters are higher in comparison. There is a general mass balance seen here, where the lower values of the residual/weathered ores is balanced by the higher values found in the surrounding waters. The waters and the residual weathering products straddle the starting composition of the source of copper.

Soils show a range of ^65^Cu values, from −0.87 to 1.10‰ (Table 3). The range is within reported values for soils derived from shale [14] and other siliciclastic rocks [4,8,9,10,11,13,31,32,33,34,35]. In general, the samples from the top 5 cm of the soils displayed relatively consistent values (0.08 ± 0.16‰; n = 17) greater than 1 km from mining activity, whereas samples taken within closer proximity and down hydrogeologic gradient on the eastern side (termed DHGES samples from this point) have a higher mean, range, and are not as consistent (0.45 ± 0.56‰; n = 15).

**Figure 4:**
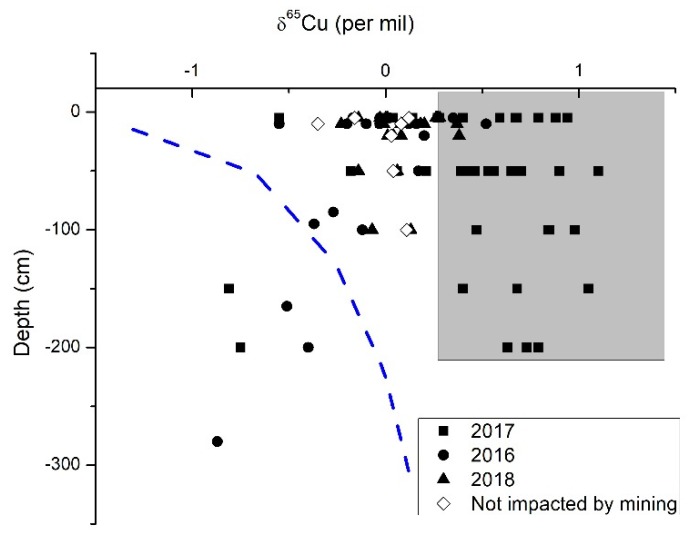
Copper isotope values of the soils plotted versus depth. Grey areas are interpreted as copper derived from mining practice. The blue dashed line indicates the hypothetical curve of soils that derived copper from the weathering of rocks that have sulfide minerals.

A distinct pattern of higher δ^65^Cu values in shallow portions of the holes, grading to lower values in deeper sections of the holes, exists for samples within about 1 km and down hydrogeologic gradient of the eastern side of the drainage (Figure 4). This pattern is the inverse of what has been documented in areas where sulfide and other silicate minerals are weathering without mining activity [10,13,14]. There is also the greatest range of Cu isotope values in this area, from ^65^Cu = −0.1 to 1.1 in the first 20 cm. In contrast, the samples from the western side of the drainage do not show this pattern and have a much tighter range of ^65^Cu = −0.2 to 0.3. The control samples taken at greater distance from the mining activity have lower Cu isotope values in shallower portions of the holes, grading to higher values in deeper sections of the holes.

## 4. Discussion

The copper isotope values presented in the waters and soils here show distinct spatial patterns, both vertically and laterally. In general, relatively higher copper isotope values are interpreted as copper sourced from mining activities, and assigning a specific value or cutoff number is complicated, as the matrix of the sample can exert controls on the mechanisms for copper isotope fractionation. This discussion focuses on how elevated copper isotope values in the waters and soils are generated and sourced from mining activities. All reservoirs that contain copper are considered in the discussion, and it is focused on a general model of mass balance.

### 4.1. The Tailings, Ores, and Waters

Tracing the source of contamination to the chalcopyrite ores hinges on a clear understanding of the starting copper isotope composition of the ores. The copper isotope variation of the starting material in skarn mineralization environments like this has been studied in several locations on earth [25,27,36]. In the ores that formed from the higher temperature fluids in skarns, the variation of copper isotope values for chalcopyrite is between ^65^Cu = −1 to 1‰. Thus, the data reported here are within the ranges reported for other skarn deposits. In the area of Tongling, the mean ^65^Cu value of −0.04 ± 0.26 is statistically tight; however, given the restricted range of sampling presented here, there is most likely a higher range of values for chalcopyrite throughout the whole mineralization system.

To trace the source chalcopyrite in the waters, a simple comparison of the water and ores provides essential insights. It has been documented that the oxidation of chalcopyrite leads to the solution becoming 1.5 per mil greater than the starting chalcopyrite [17,21,37,38]. This relationship has also been seen in areas near Tongling [26]. In this instance, as seen in waters within the 6 km radius, the mean ^65^Cu is 1.8 compared to a starting mean of the ore of −0.04. The difference between the two means is nearly identical to the experimental values determined for leaching chalcopyrite. Two outliers of >5 per mil exist in the dataset and could be related to an unknown point source not constrained in this dataset. Of course, there is large scatter in the data of the waters, and the mean value may not be ideal statistically; however, the waters are clearly higher than the starting ores and are within the same order as the laboratory experimentation and other areas known for chalcopyrite sourced by oxidative weathering.

Importantly, the loss of the heavier copper from the source materials is recorded in the tailings (Figure 3). The tailings values are less than the starting ores and show a systematic lighter copper isotope value in shallower portions of the hole. This could indicate that the tailings exposed to the surface have been oxidized more and lost more copper. The increase in values down hole suggest this as a possible explanation for the variation. This pattern has been clearly documented in soils and leach caps, where greater exposure to oxidation leads to the pattern seen [14,39].

Does all of the copper released in the area originate in the tailings? This is an important question, which these data cannot resolve. Chalcopyrite could exist within the mining operation, and mining processes introduce oxidative solutions and gases that could aid in weathering chalcopyrite from within the underground operations. Fluids derived from this activity could also add to the copper isotope signals recorded in subsurface and surface waters. Thus, the oxidation of one reservoir, or a combination of the tailings and actively weathering ores, can explain the copper isotope data of the tailings, ores, and waters.

The water data clearly show that copper derived from the Tonlging mining practice at Shizishan does not travel into the Yangtze River. Vance and Archer [40] show that the copper isotope composition of the Yangtze about 50 km upstream of this location has a copper isotope value of 1.3‰. The main drainage from the Tongling ore fields here has a ^65^Cu of only 0.3 and does not have copper that originated from the mining practice at Shizishan, nor would this input show copper derived from mining. The high value of the Yangtze River is important to note, and why this value is the highest reported by Vance and Archer could be related to other copper inputs within the system.

### 4.2. The Soils

The DHGES samples display the greatest ranges of copper isotope values and represent copper incorporated from mining activity into the soils. The source must be the mining operations, as the control samples, along with the set of samples taken on the opposite side of the mining operation, display a much smaller range of copper isotope values. The larger copper isotope values in the soils near the mining operation could potentially be explained by multiple inter-related processes. The discussion incorporates several potential explanations and argues that the most likely cause for the higher values is related to groundwater flow that contains heavier copper which subsequently adsorbed onto mineral grains in the soils. Groundwater traveling through the soils in the vadose zone or fluxing through the migration of the water table could carry the metal to produce this signature. Water was encountered in the holes at depths greater than 50 cm.

If no copper was derived from other sources during the weathering of a limestone rock that contains sulfide minerals, the profiles of the soils would most likely display the lightest copper isotope values at the top of the hole, and these values would generally increase to the starting copper isotope value of the source at depth. The pattern has been documented in weathering of silicate rocks with sulfide minerals [4,14,39]. This pattern is displayed as a blue dashed line in Figure 4 and represents what the combination of biogeochemical reactions would generate with no mining activity. The control samples and TS 1, 3, 4, 6, and 8 show a general increase in Cu isotope values with depth and could be linked to weathering of sulfide minerals. As shown in Reference [14], the progressive weathering of black shale with sulfide minerals generates isotopically lighter source values near the surface in the older soils in comparison to heavier values in newly developed soils near the rock-soil boundary. The diamond symbols in Figure 4 show this general trend. However, the ranges are tighter than seen in silicate rocks previously reported.

**Figure 5:**
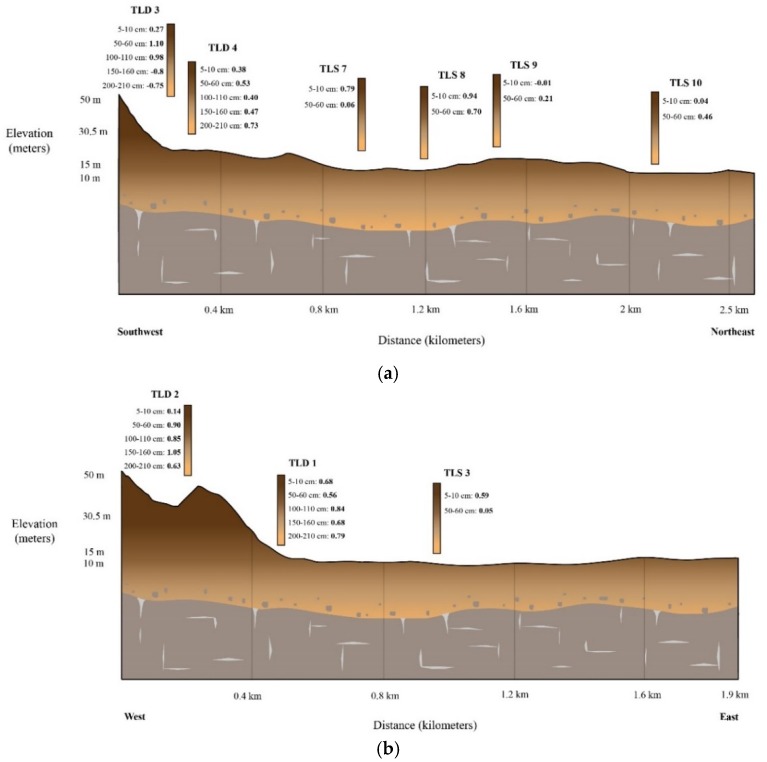

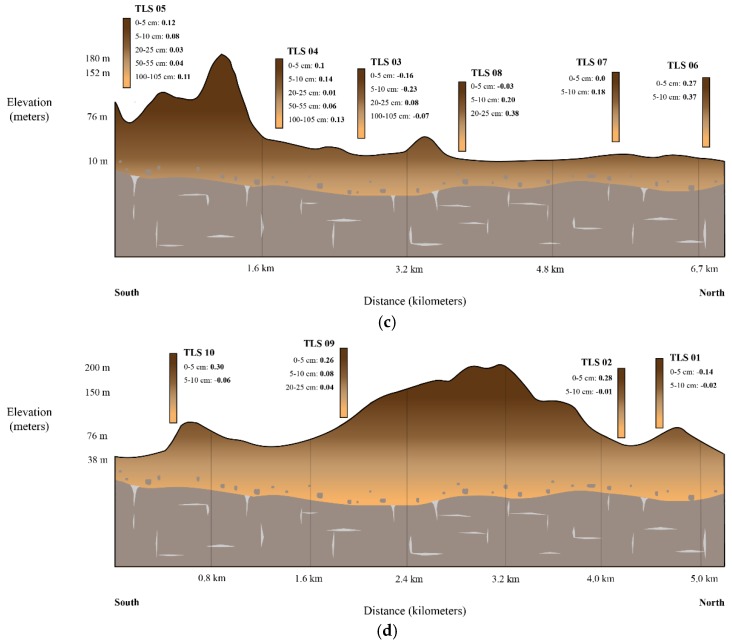
Soil cross sections for the samples to observe vertical variations of copper isotope values. Depth to bedrock is not to scale and was simply added to the images. The profiles correspond to the dashed cross section lines seen in Figure 1: (**a**) yellow 1; (**b**) green 2; (**c**) grey 4; (**d**) black.

Note that when comparing the profiles for each hole in the cross sections in Figure 5, none of the DHGES samples within 1.5 km of the mining activity have this profile. In fact, the profile is inversed, where the higher values are found in upper portions of the hole and lower values are found deeper. Therefore, weathering of sulfide minerals during soil generation does not produce the copper isotope profiles seen in the DHGES samples.

Either a heavy copper isotope source being added to the soils, or lighter copper being preferentially removed, could explain the different copper isotope patterns in the DHGES samples. Multiple studies have shown that the organic rich layer in the first 5 cm of soils have low copper isotope values due to the activity of organic materials which favor the lighter copper isotope [4,41,42]. Slightly lower values in the first 5 cm are found in multiple profiles and could be explained by increased organic materials. Another potential explanation for the lower values found in the first 5 cm of all holes in the area could be related to an airborne source from the smelter. Note that the copper isotope composition of the ores matches the signature found in all holes in the first 0–5 cm. To further understand the source of copper in the upper several cm of the soil profiles, samples of dust or filter type fungi that collect airborne particles are needed.

The addition of heavy copper by sorption of copper onto the clay minerals most likely explains the pattern for these DHGES samples. In buffered/higher pH solutions, as present in the soils derived from limestones, copper has been shown experimentally, and in the field, to readily absorb onto Fe-oxides that are the product of sulfide weathering [42,43,44]. The adsorption process has been demonstrated to produce a +0.2 per mil for ^65^Cu (Fe-oxide-solution) onto the Fe-oxide phases [8,9,13,19,45]. Thus, the heavier copper isotope signatures found in the soils most likely results from groundwaters that carry higher copper isotope values (due to the oxidation of copper from sulfide phases) in combination with the heavier isotope fractionated onto the Fe-oxides during adsorption. This twofold process, working in concert, results in heavier copper isotope values in the soils sourced from the oxidation of sulfides related to mining activity.

Empirical evidence for this process exists in two different observations of the dataset. First, the copper isotope values of the surfaces waters become significantly lighter from the source down hydrogeological gradient (from ^65^Cu of 5.5‰ to 0.6‰). The adsorption process occurring between the soil-groundwater as it flows down gradient could cause the decrease in values. Second, as seen in the vertical profiles, the heavier copper isotope values are in deeper parts the holes that are closer to the tailings source. The values appear to form a wedge shape in profiles A and B in Figure 5, of heavier values in shallower parts of the holes which taper away from the tailings. The tapered values could originate from the groundwater flow path.

### 4.3. Migration of Copper as Identified by Copper Isotopes in Tongling

The copper isotope composition of the ores, tailings, waters, and soils provide a general model for how and where copper is currently moving in Tongling due to mining activities. The process starts with the oxidation of sulfide-bearing minerals that possess copper which have been exposed in the tailings and through the mining operation. This reaction results in solutions that have isotopically heavier copper in comparison to all other geologic materials in the area. The heavier isotopic composition in the fluids records this interaction in both the surface waters and soils. The heavier copper in the waters is transferred into the soils via adsporption processes, and the copper isotopic signal persists in the waters down hydrogeological gradient.

Proof of these interactions exists in the general copper isotopic mass balance within reservoirs which house Cu. The oxidation reactions that resulted in the release of ^65^Cu are found in the waters, which are distinctly heavier in about a 6 km radius of the mining activity, and in the residual tailings, which are isotopically lighter. The soils nearby the mine serve to remove copper from the surface and subsurface water by an adsorption process which takes place within 1.5 km from the mine.

## 5. Conclusions

The results presented show that copper derived from mining practices cause a dispersion of metal into the surrounding areas approximately 6 km down hydrogeological gradient, because the heavier copper isotope values diminish beyond this distance. Copper sourced from the Shizshan deposit does not appear to contribute copper to the Yangtze River due to the lack of heavier copper isotope values upstream in the major tributary feeding the Yangtze. Copper derived from sulfide oxidation appears to be introduced into the surrounding soils within closer proximity of the mining operation. Elevated copper isotope values in soils proximal to the deposit show this relationship, whereas soils greater than 1 km away do not possess a heavier copper isotope signature.

This study demonstrates that the Cu isotope composition of tailings, ores, soils, and waters from areas near copper sulfide mines can be used to track the source of Cu migration in the area. The data provide a means to fingerprint copper sources in materials that may possess relatively low concentrations of copper. The technique appears to be a robust means to monitor copper dispersion into the environment from actively weathering copper minerals.

## Figures and Tables

**Table 1:** Copper isotope values of ores, where cpy = chalcopyrite and py = pyrite.

Sample	Mineral Phase	^65^Cu (per mil)
TLJG-49	cpy	0.08
TLJG-51	cpy	−0.01
TLJG-53.2	cpy	0.01
TLJG-54.1	cpy	0.28
TLJG-54.2	cpy	−0.29
TLJG-55	cpy	−0.13
TLJG-57.1	cpy	−0.21
TLJG-57.2	cpy	0.57
TLJG-64	cpy	0.03
TLJG-42	py	0.82
TLJG-52	py	0.11
TLJG-60	py	−0.31
TLJG-63	py	1.83

**Table 2:** Copper isotope values for the waters.

Water Sample	Year	^65^Cu (per mil)
TLW03	2016	0.38
TLW02	2016	0.74
TLW01	2016	0.34
TLW05	2016	0.38
TLW13	2016	1.79
TLW14	2016	6.90
TLW15	2016	0.36
TLW11	2016	0.36
TLW08	2016	−0.44
TLW09	2016	0.60
TLW07	2016	0.50
TLW06	2016	5.49
TLW17	2016	−0.13
TLW16	2016	1.20
1 W	2018	0.99
2 W	2018	0.99
3 W	2018	0.67
4 W	2018	1.01
5 W	2018	1.89
6 W	2018	1.24
7 W	2018	1.27

**Table 3:** Copper isotope values of the soils. Asterisks indicate soils not thought to be impacted by mining practice.

Sample	Year	Depth (cm)	^65^Cu (per mil)
01 01	2016	60	−0.34
01 02	2016	25	−0.32
01 03	2016	10	−0.66
02 01	2016	5	0.35
02 02	2016	10	0.52
02 03	2016	20	0.20
02 04	2016	50	0.17
02 05	2016	100	−0.12
02 07	2016	200	−0.40
02 08	2016	280	−0.87
03 01	2016	5	−0.03
03 02	2016	10	0.12
04 01	2016	10	−0.10
04 02	2016	10	−0.55
05 01	2016	5	0.02
05 02	2016	10	−0.20
06 01	2016	5	0.01
06 02	2016	10	−0.03
06 03	2016	95	−0.37
06 04	2016	165	−0.51
07 01 *	2016	5	−0.16
07 02 *	2016	10	−0.35
08 01	2016	5	−0.17
08 02	2016	10	0.16
08 03	2016	85	−0.27
TLD 1a	2017	5	0.68
TLD 1b	2017	50	0.56
TLD 1c	2017	100	0.84
TLD 1d	2017	150	0.68
TLD 1e	2017	200	0.79
TLD 23	2017	100	0.85
TLD 2a	2017	5	0.14
TLD 2b	2017	50	0.90
TLD 2d	2017	150	1.05
TLD 2e	2017	200	0.63
TLD 03a	2017	5	0.27
TLD 03b	2017	50	1.10
TLD 03c	2017	100	0.98
TLD 03d	2017	150	−0.81
TLD 03e	2017	200	−0.75
TLD 04a	2017	5	0.28
TLD 04b	2017	50	0.53
TLD 04c	2017	100	0.47
TLD 04d	2017	150	0.40
TLD 04e	2017	200	0.73
TLS 05b	2017	50	−0.18
TLS 06a	2017	5	0.40
TLS 06b	2017	50	0.65
TLS 07a	2017	5	0.79
TLS 07b	2017	50	0.06
TLS 08a	2017	5	0.94
TLS 08d	2017	50	0.70
TLS 09a	2017	5	−0.01
TLS 09b	2017	50	0.21
TLS 10b	2017	50	0.46
TLS 1a	2017	5	0.88
TLS 1b	2017	50	0.45
TLS 1b	2017	50	0.39
TLS 2a	2017	5	−0.55
TLS 2b	2017	50	0.06
TLS 3a	2017	5	0.59
TLS 3b	2017	50	0.05
TLS 4a	2017	5	0.67
TLS 4b	2017	50	0.44
TLS0 10a	2017	5	0.04
TLS01A	2018	5	−0.14
TLS01B	2018	10	−0.02
TLS02A	2018	5	0.28
TLS02B	2018	10	−0.01
TLS03A	2018	5	−0.16
TLS03B	2018	10	−0.23
TLS03C	2018	20	0.08
TLS03E	2018	100	−0.07
TLS04A	2018	5	0.01
TLS04B	2018	10	0.14
TLS04C	2018	20	0.01
TLS04D	2018	50	0.06
TLS04E	2018	100	0.13
TLS05A *	2018	5	0.12
TLS05B *	2018	10	0.08
TLS05C *	2018	20	0.03
TLS05D *	2018	50	0.04
TLS05E *	2018	100	0.11
TLS06A	2018	5	0.27
TLS06B	2018	10	0.37
TLS07A	2018	5	0
TLS07B	2018	10	0.18
TLS08A	2018	5	−0.03
TLS08B	2018	10	0.2
TLS08C	2018	20	0.38
TLS09A	2018	5	0.26
TLS09B	2018	10	0.08
TLS09C	2018	20	0.04
TLS10A *	2018	5	0.3
TLS10B *	2018	10	−0.06
TLS03D	2018	50	−0.14
